# Validation study for the hypothesis of internal mammary sentinel lymph node lymphatic drainage in breast cancer

**DOI:** 10.18632/oncotarget.9634

**Published:** 2016-05-26

**Authors:** Bin-Bin Cong, Peng-Fei Qiu, Yan-Bing Liu, Tong Zhao, Peng Chen, Xiao-Shan Cao, Chun-Jian Wang, Zhao-Peng Zhang, Xiao Sun, Jin-Ming Yu, Yong-Sheng Wang

**Affiliations:** ^1^ School of Medicine and Life Sciences, University of Jinan and Shandong Academy of Medical Sciences, Jinan, Shandong, China; ^2^ Breast Cancer Center, Shandong Cancer Hospital Affiliated to Shandong University, Jinan, Shandong, China; ^3^ Department of Radiotherapy, Shandong Cancer Hospital Affiliated to Shandong University, Jinan, Shandong, China

**Keywords:** breast cancer, internal mammary, sentinel lymph node biopsy, visualization rate, indocyanine green

## Abstract

According to axilla sentinel lymph node lymphatic drainage pattern, we hypothesized that internal mammary sentinel lymph node (IM-SLN) receives lymphatic drainage from not only the primary tumor area, but also the entire breast parenchyma. Based on the hypothesis a modified radiotracer injection technique was established and could increase the visualization rate of the IM-SLN significantly. To verify the hypothesis, two kinds of tracers were injected at different sites of breast. The radiotracer was injected with the modified technique, and the fluorescence tracer was injected in the peritumoral intra-parenchyma. The location of IM-SLN was identified by preoperative lymphoscintigraphy and intraoperative gamma probe. Then, internal mammary sentinel lymph node biopsy (IM-SLNB) was performed. The fluorescence status of IM-SLN was identified by the fluorescence imaging system. A total of 216 patients were enrolled from September 2013 to July 2015. The overall visualization rate of IM-SLN was 71.8% (155/216). The success rate of IM-SLNB was 97.3% (145/149). The radiotracer and the fluorescence tracer were identified in the same IM-SLN in 127 cases, the correlation and the agreement is significant (*Case-base,* r_s_=0.836, *P*<0.001; *Kappa*=0.823, *P*<0.001). Different tracers injected into the different sites of the intra-parenchyma reached the same IM-SLN, which demonstrates the hypothesis that IM-SLN receives the lymphatic drainage from not only the primary tumor area but also the entire breast parenchyma.

## INTRODUCTION

Internal mammary lymph node (IMLN) metastasis has a similar prognostic importance as axillary lymph node (ALN) involvement in breast cancer patients [1–3]. Internal mammary sentinel lymph node biopsy (IM-SLNB) was a minimally invasive technique for the efficient evaluation of the status of internal mammary sentinel lymph nodes (IM-SLN) with high safety and feasibility [4, 5]. The success rate of IM-SLNB has reached 60-100% with minimal or no changes in operative time [4–7], but the visualization rate of IM-SLN was low (average 13%, range 0-37%) by the traditional injection technique [6–8].

According to the axilla sentinel lymph node (ASLN) lymphatic drainage pattern (i.e., ASLN receives lymphatic drainage from not only the primary tumor area, but also the entire breast) [9, 10], we hypothesized that IM-SLN receives lymphatic drainage from not only the primary tumor area, but also the entire breast parenchyma [8]. Based on the hypothesis, a modified radiotracer injection technique has been established which increased the visualization rate of the IM-SLN significantly [8]. The hypothesis needs to be demonstrated. To avoid more injury by complete IMLN dissection following IM-SLNB, an alternative validation study for the hypothesis was performed: two different tracers were injected in different sites of the intra-parenchyma to observe whether they could reach to the same IM-SLN.

## RESULTS

### Characteristics of IM-SLNB

The clinically pathological characteristics of the 216 enrolled patients are presented in Table 1. The detection rate of ASLN was 98.6% (213/216). The overall visualization rate of IM-SLN detected by preoperative lymphoscintigraphy and gamma probe was 71.8% (155/216). 96.1% (149/155) of them received IM-SLNB. The success rate of IM-SLNB was 97.3% (145/149). The data on clinical outcome of the patients underwent IM-SLNB show in Table 2. In 12 patients underwent breast conserving surgery, 5 cases who were identified the location of primary tumor could not reach IM-SLNB had to be made an extra incision in the skin to reach IM-SLNB.

**Table 1:** Descriptive characteristics of eligible patients (N = 216)

Characteristic	No.		%
Age (years)			
Median		50	
Range		27~79	
≤50	119		55.1
>50	97		44.9
BMI			
Median		24.1	
Range		17.2~33.5	
Tumor size			
Tis	16		7.4
T1	99		45.8
T2	79		36.6
T3	22		10.2
Tumor location			
UOQ	92		42.6
LOQ	25		11.6
UIQ	48		22.2
LIQ	5		2.3
Central	46		21.3
Tumor type			
Ductal	187		86.6
Lobular	8		3.7
Mixed	5		2.3
Other	16		7.4
Radiotracer intensity (MBq)			
Median		36	
Radiotracer volume (mL/point)			
Median		0.5	
Intervals from injection to SLNB (h)			
2~5	89		41.2
16~22	127		58.8

Abbreviations: *BMI* body-mass-index, *UOQ* upper outer quadrant, *LOQ* lower outer quadrant, *UIQ* upper inner quadrant, *LIQ* lower inner quadrant.

**Table 2:** Clinical outcome of patients who underwent IM-SLNB (N = 145)

Characteristic	No.	%
T Stage		
Tis	9	6.2
T1	70	48.3
T2	57	39.3
T3	9	6.2
N Stage		
N0	70	48.3
N1	57	39.3
N2	7	4.8
N3	11	7.6
ER		
Positive	101	69.7
Negative	44	30.3
PR		
Positive	98	67.6
Negative	47	32.4
HER-2		
Positive	44	30.3
Negative	101	69.7
Type of surgery		
Lumpectomy+ASLNB	9	6.2
Lumpectomy+ALND	3	2.1
Mastectomy+ASLNB	93	64.1
Mastectomy+ALND	40	27.6
Radiotherapy		
WBI	7	4.8
WBI+RNI	5	3.5
PMRT+RNI	79	54.5
No	54	37.2
Chemotherapy		
Yes	121	83.4
No	24	16.6

Abbreviations: *ER* estrogen receptor status, *PR* progesterone receptor status, *HER-2* human epidermal growth factor receptor-2, *WBI* whole breast irradiation, *RNI* regional node irradiation, *PMRT* postmastectomy radiotherapy.

In patients who performed IM-SLNB successfully, a total of 279 lymph nodes were removed, the median number of IM-SLNs was 2 (range 1-4 nodes). The IM-SLNs were located in the first (5.4%, 15/279), second (46.2%, 129/279), third (40.5%, 113/279) and forth (7.9%, 22/279) intercostal space. All positive IM-SLNs were in the second (61.1%, 11/18) and the third (38.9%, 7/18) intercostal space. 54.1% (151/279) of IM-SLN was found in the outside of the internal mammary vessels and 45.9% (128/279) was in the inside. Details of IM-SLN mapping and biopsy are shown in Table 3. The IM-SLN involvement rate was 8.1% (7/86) in patient with clinically axillary node negative patients and 18.6% (11/59) in positive patients respectively. All patients with positive IM-SLN received regional nodal irradiation to IMLNs. The clinical, pathological and treatment details of these patients were shown in Table 4. In patients with ≥4 positive axillary lymph nodes, regional nodal irradiation to IMLNs had been avoided in 50.0% cases (9/18) with negative IM-SLN. In patients with 1-3 positive axillary lymph nodes, regional nodal irradiation to IMLNs might be avoided in 91.2% cases (52/57) with negative IM-SLN.

**Table 3:** Details of IM-SLN mapping and biopsy

Characteristic	No.		%
IM-SLN map+	155		71.8 (155/216)
Pt. performed IM-SLNB	149		96.1 (149/155)
Success rate of IM-SLNB	145		97.3 (145/149)
Total No. of IM-SLN		279	
Median		2	
Range		1~4	
IM-SLN metastatic	18		12.4 (18/145)
IM-SLNB time (min)			
Median		10	
Range		3~55	
IM-SLN size (mm)			
Median		5	
Range		3~12	

**Table 4:** The clinical, pathological and treatment details of patients with positive IM-SLN

No.	Tumor location	T stage	No. of positive ALN	N stage without IM-SLN	No. of positive IM-SLN	N stage with IM-SLN	Finally stage	Chemo-Therapy	Radio-Therapy
1	UOQ	T2	0	pN0	2	pN1b	IIA→IIB	Yes	No→Yes
2	UIQ	T2	2	pN1a	1	pN1c	IIB (no change)	Yes	? →Yes
3	Central	T2	14	pN3a	1	pN3b	IIIC (no change)	Yes	Yes
4	UOQ	T2	9	pN2a	1	pN3b	IIIA→IIIC	Yes	Yes
5	UIQ	T1c	2	pN1a	1	pN1c	IIA (no change)	Yes	? →Yes
6	UOQ	T2	1	pN1a	1	pN1c	IIB (no change)	Yes	? →Yes
7	UIQ	T1a	0	pN0	1	pN1b	IA→IIA	No→Yes	No→Yes
8	UOQ	T2	9	pN2a	2	pN3b	IIIA→IIIC	Yes	Yes
9	LIQ	T2	5	pN2a	1	pN3b	IIIA→IIIC	Yes	Yes
10	UOQ	T1a	3	pN1a	1	pN1c	IIA (no change)	Yes	? →Yes
11	UIQ	T2	0	pN0	1	pN1b	IIA→IIB	Yes	No→Yes
12	UOQ	T3	13	pN3a	1	pN3b	IIIC (no change)	Yes	Yes
13	Central	T1c	1	pN1a	1	pN1c	IIA (no change)	Yes	? →Yes
14	UOQ	T2	13	pN3a	1	pN3b	IIIC (no change)	Yes	Yes
15	Central	T2	11	pN3a	1	pN3b	IIIC (no change)	Yes	Yes
16	UOQ	T2	20	pN3a	1	pN3b	IIIC (no change)	Yes	Yes
17	UOQ	T2	5	pN2a	1	pN3b	IIIA→IIIC	Yes	Yes
18	UIQ	T1c	0	pN0	1	pN1b	IA→IIA	No→Yes	No→Yes

Abbreviations: *UOQ* upper outer quadrant, *UIQ* upper inner quadrant, *LIQ* lower inner quladrant, *?* radiotherapy is controversy.

### Correlation and agreement between the radiotracer and the fluorescence tracer

In the validation study, 145 patients underwent IM-SLNB successfully, of which 127 patients identified the radiotracer and the fluorescence tracer reached to the same IM-SLN, 18 patients were detected only the radiotracer positive IM-SLN (Table 5). Accordingly, the radiotracer and the fluorescence tracer in the same IM-SLN showed a strong correlation coefficient at 0.836 (*Case-base*, r_s_≥0.5, *P*<0.05). The degree of agreement between the radiotracer and the fluorescence tracer was *Kappa*=0.823 (very good), showing high degree of agreement between the two tracers (*Kappa*>0.8, *P*<0.05).

**Table 5:** Different tracers identified in IM-SLN

Tracers map	Radiotracer+	Radiotracer-	Total
**Fluorescence tracer+**	127	0	127
**Fluorescence tracer-**	18	71	89
**Total**	145	71	216

### Complications

No serious bleeding and pain was found after using the modified radiotracer injection technique in all patients. A total of 2 patients were found with minor generalized skin reactions which occurred after injection of indocyanine green (ICG) during the surgery. A small pleural lesion (≤ 2 mm) was noted intraoperatively in 1.4% cases (2/145) and no pneumothorax was seen postoperatively on chest X-rays. Intraoperative bleeding from the internal mammary artery occurred in 3.4% cases (5/145), and was successfully resolved. There were no postoperative complications and reactions after the two-week following up, and no increase of days in hospital stay from this procedure.

## DISCUSSION

IMLN metastases have been demonstrated to occur in 28-52% of ALN positive patients and 5-17% of ALN negative patients [2, 11, 12]. In patients with a negative axilla, a positive IMLN portends a similar prognosis as ALN positive patients, impacting both recurrence and survival [1–3]. The results of the MA.20 showed that the addition of regional nodal irradiation (including IMLNs) to whole-breast irradiation reduced the rate of breast-cancer recurrence in patients with node-positive or high-risk node-negative breast cancer [13]. The EORTC 22922/10925 study found that regional nodal irradiation improved the rates of disease-free and distant disease-free survival and reduced the rate of death from breast cancer among patients with early-stage breast cancer [14]. Furthermore, the DBCG-IMN study identified that IMLNs irradiation increased overall survival in patients with early-stage node-positive breast cancer [15]. The 2016 National Comprehensive Cancer Network Breast Cancer Clinical Practice Guidelines recommend radiotherapy to IMLNs for patients with ≥4 positive ALNs (category 1), and strongly consider radiotherapy to IMLNs for patients with 1-3 positive axillary nodes (category 2A), both after mastectomy and lumpectomy [16]. However, low-risk did not mean IMLN negative and high-risk did not mean IMLN metastases [17]. Studies of extended radical mastectomy reported that 36.8%-46.2% patients with ≥4 positive ALNs and 18.8%-26.7% patients with 1-3 positive ALNs identified IMLN metastases, and negative IMLN was found in about 70% patients with ≥4 positive ALNs [1, 18–20]. Thus, these inclusion criteria might induce over- and under-treatment. Up to now, there have no reasonable methods to detect out metastasis in IMLN exactly. But IM-SLNB via intercostal space could make it possible—tailored IMLNs radiotherapy and minimally invasive staging [17]. Even though breast cancer staging has incorporated IM-SLNB concept since the 6^th^ edition of the American Joint Committee on Cancer, IM-SLNB has not been performed routinely [21]. The studies of IM-SLNB showed that the success rate of IM-SLNB has reached 60-100% with minimal or no changes in operative time [4–7], but the visualization rate of IM-SLN was low [6–8], which has been the restriction for both clinical study and daily practice of IM-SLNB (Table 6).

**Table 6:** Currently studies results of IM-SLNB

First author	Year	Case	IM-SLN visualization % (Case)	IM-SLNB success % (Case)	IMLN positive % (Case)
Dupont [22]	2001	1470	2.4 (36)	100 (36)	13.9 (5)
van der Ent [23]	2001	256	25.4 (65)	63.1 (41)	26.8 (11)
Paganelli [24]	2002	400	15.8 (63)	93.7 (59)	8.5 (5)
Estourgie [25]	2003	691	21.7 (150)	86.7 (130)	16.9 (22)
Farru's [26]	2004	225	8.8 (20)	70.0 (14)	14.3 (2)
Hong [27]	2005	979	14.1 (138)	100 (138)	18.1 (25)
Carcoforo [28]	2006	741	14.4 (107)	60.7 (65)	15.4 (10)
Leidenius [29]	2006	984	14.0 (138)	87.7 (121)	14.9 (18)
Madsen [30]	2007	506	21.5 (109)	78.0 (85)	23.5 (20)
Heuts [5]	2009	1008	19.4 (196)	70.9 (139)	22.3 (31)
Bourre [31]	2009	622	28.0 (174)	92.5 (161)	11.1 (18)
Postma [4]	2012	493	24.1 (119)	72.3 (86)	16.3 (14)
Ozmen [32]	2015	890	8.1 (72)	100 (72)	13.9 (10)

Clinical studies found that superficial injection (intradermal, subdermal, periareolar, and subareolar) of radiotracer was hard to identify IM-SLN but intraparenchymal injection (peritumoral, intratumoral, or subtumoral) was more reliable [33–38]. These results suggest that the dermal and subdermal lymphatic flow is rarely directed to IMLNs, whereas some intraparenchymal lymphatic flow is directed to IMLNs. Anatomy study described that the breast parenchyma has extensive lymphatic network and has rich anastomoses with the superficial cutaneous lymph plexus of the developing skin [39]. It is considered that radiotracer, wherever injected, could flow to the same ASLN. That means ASLN receive the lymphatic drainage from not only the primary tumor area, but also the entire breast organ [10]. Based on this concept, the hypothesis of IM-SLN was supposed, which IM-SLN receives lymphatic drainage from not only the primary tumor area, but also the entire breast parenchyma. According to the hypothesis, a modified technique (periareolar intra-parenchyma, high volume, and ultrasonographic guidance) was formulated, which could significantly increase the preoperative visualization rate of the IM-SLN without lowering ASLN visualization rate [8, 38]. The detection rate of IM-SLN was 71.1% in the modified technique group compare to 15.5% in the traditional technique group (*P*<0.001), and the detection rate of ASLN was similar in both groups (98.9% versus 98.3%, P=0.712) [38]. The visualization number of IM-SLN was no difference between the modified technique group and the traditional tracer technique in our pilot study (*P*=0.692). Also, the preoperative visualization number of IM-SLN was largely in accordance with other studies used tradition technique (peritumoral intraparenchymal injection) [40].

However, the radiotracer was not injected in peritumoral intra-parenchyma but in periareolar intra-parenchyma with the modified technique based on the hypothesis. The question arises as to whether all nodes detected by the method should be considered as “true” IM-SLN or whether some of them are actually “second-tier” IMLN. The accuracy of the modified technique has been confirmed by our team at the previous study [41]. The hypothesis of ASLN lymphatic drainage pattern was proved with subsequent axillary lymph node dissection (ALND). As the extended radical mastectomy (included all IMLNs resection) has been abandoned since 1960s [42, 43], the hypothesis of IM-SLN lymphatic drainage pattern cannot be validated by this way. Now, another method was used to validate the IM-SLN lymphatic drainage hypothesis. The ICG fluorescence tracer is a safe and effective method for SLNB in breast cancer with acceptable sensitivity and specificity comparable to conventional methods [44–46]. In our breast cancer center, it has been compared with the combined method (blue dye with radiotracer) in identifying ASLN. It showed that all ASLN identified by combined method also were ICG fluorescence positive and non-sentinel lymph nodes were ICG negative after ALND (n= 69, P<0.05). The internal mammary lymph nodes commonly receive less than 25% of the total lymph from the breast [47]. Due to little volume of ICG tracer is difficulty to detect by the fluorescence imaging system, it is hard to find IM-SLN by this tracer. But IM-SLN can be detected by radiotracer with the modified radiotracer injection technique and performed biopsy. In the validation study, the ICG fluorescence tracer was injected intraparenchymally at the peritumoral and the radiotracer was injected with the modified technique. By this method, to identify different tracers injected in different sites could reach to the same IM-SLN. After IM-SLNB the status of IM-SLN was identified by intraoperative gamma probe and fluorescence imaging system. The radiotracer and the fluorescence tracer in the same IM-SLN showed a strong correlation coefficient at 0.836 (*Case-base*, r_s_>0.5, *P*<0.05). The degree of agreement between the radiotracer and the fluorescence tracer was *Kappa*=0.823 (very good), showing high degree of agreement between the two tracers (*Kappa*>0.8, *P*<0.05). It showed that the lymphatic drainage from different location of the breast (the primary tumor, the subareolar plexus) reached to the same IM-SLN, which means that IM-SLN receives lymphatic drainage from not only the primary tumor area but also the entire breast parenchyma. That means the hypothesis of IM-SLN lymphatic drainage pattern was demonstrated. Furthermore, IM-SLN detected by the modified technique could reflect the real lymphatic drainage of the whole breast parenchyma. In other words, the modified technique can detect the “true” sentinel node in the internal mammary chain. In the study, the results of the metastases site and the number of IM-SLNs were in accordance with the past study of extended radical mastectomy, which could reflect the accuracy of IM-SLNB indirectly [48–50]. There were no serious adverse events or reactions after the radiotracer injected guiding by the modified injection technique.

In sum, different tracers injected into the different sites of the intra-parenchyma reached the same IM-SLN, which demonstrates the hypothesis that IM-SLN receives the lymphatic drainage from not only the primary tumor area but also the entire breast parenchyma.

## PATIENTS AND METHODS

### Study design and patients

From September 2013 to July 2015, 216 patients with core biopsy proved invasive breast cancer scheduled to receive preoperative tracers injection, who agreed with performing IM-SLNB as part of their breast cancer surgery, were recruited to the IM-SLNB study. Patients with previous invasive breast cancer, hypersensitivity to iodine or ICG, hyperthyroidism and patients who were either pregnant or lactating were excluded from the study.

**Figure 1:**
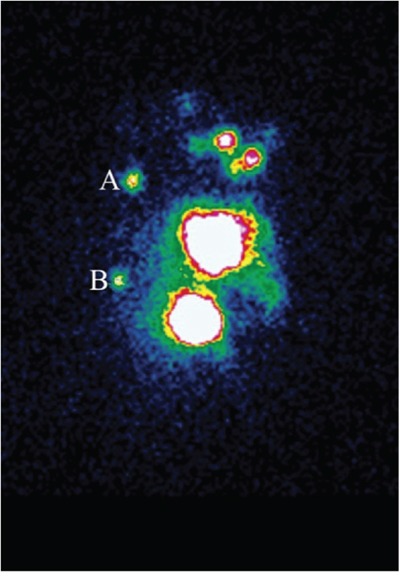
Preoperation lymphoscintigram with radiotracer Hotspots are evidently shown in both the second intercostal space **A.** and the fourth intercostal space **B.** in patient with left-sided breast cancer.

**Figure 2:**
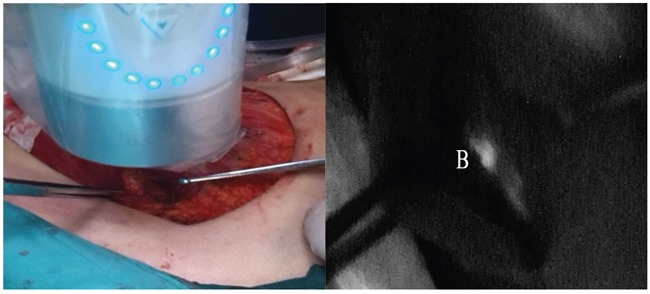
Intraoperative IM-SLNB identified the location of IM-SLN in the fourth intercostal space The fluorescence imaging system showed the IM-SLN fluorescence tracer positive **B.**

Two different kinds of tracer were injected at different sites in this validation study. The radiotracer (1.0-1.2ml 9.25-18.5MBq ^99m^Tc-labeled sulfur colloid) was injected with the modified radiotracer injection technique. It was injected into intra-parenchyma at the 6 and 12 o'clock positions 0.5-1.0cm from the areola guided by ultrasound (ALOKA-SSD-5000, ALOKA, Tokyo, Japan) 3-18h before surgery [8, 38]. The fluorescence tracer (1.0ml 0.5% ICG) was injected in the peritumoral intra-parenchyma guided by ultrasound just before the beginning of the peration. The radioactive IM-SLNs were detected by preoperative lymphoscintigraphy (Toshiba GCA 901AHG, Toshiba Corporation, Tokyo, Japan) (Figure 1) 30min before the surgery and gamma probe (Neoprobe, Neo2000 gamma detection system, Johnson & Johnson New Brunswick, NJ, USA) during the surgery. IM-SLNB was performed for patients with the radioactive IM-SLNs. After IM-SLNB, the fluorescent status of IM-SLN was identified with the fluorescence imaging system (Ming De, MD fluorescence imaging system, Langfang, People's Republic of China) (Figure 2). The number and the status of IM-SLNs were recorded to identify the IM-SLN visualized rate and the concordance rate of the radiotracer and the fluorescence tracer. All IM-SLNs were assessed by routine pathology.

The study was conducted within a single institute (Breast Cancer Center, Shandong Cancer Hospital Affiliated to Shandong University). All patients gave informed consent to participate in the study which had approval from the Shandong Cancer Hospital Affiliated to Shandong University Research Ethics Board (No. SDTHEC20130324). Any immediate toxicity following injection of ICG and any adverse events during the study period were recorded during surgery and at the two-week follow up appointment.

### Internal mammary sentinel lymph node biopsy

During the operation, IM-SLNB was performed for patients with radioactive IM-SLNs via the intercostal space. Through the mastectomy incision or modified radical mastectomy incision access to the location of IM-SLNs under the guidance of intraoperative gamma probe. Then the pectoral major fascia muscle fibers were separated to expose the relevant intercostal space. Next, the external and internal intercostal muscles were divided transversely in its middle. To avoid injury to the anterior intercostal vessels, the division should be located between the two costal cartilages or along the superior costal border. IM-SLNs located in other intercostal spaces were removed by the same method. A postoperative chest X-ray was performed in case of accidental pleural lesion.

In patients who accepted breast conserving surgery, if the location of primary tumor could not reach IM-SLNB, an extra incision (2.5-3.0cm) in the skin has to be made.

### Histopathology of sentinel lymph nodes

All SLNs underwent pathological evaluation according to local protocol including serial sectioning at 2.0-3.0mm followed by routine staining with Haematoxylin and Eosin.

### Statistical analysis

The data were analyzed with the SPSS 17.0 software package. Chi-square-test or Fisher's-exact-test was performed to compare the visualization rates among the groups. The correlations between the radiotracer and the fluorescence tracer in the same IM-SLN were calculated using the Spearman rank correlation coefficient. The criteria for judging the size of the correlation coefficient were applied: correlations<0.30 are considered minor, correlations between 0.3-0.49 are considered medium, and ≥0.5 are considered strong. Cohen's kappa statistic was used to determine inter-examiner agreement. According to Altman's guidelines, it is poor when kappa scores ≤0.20, fair when kappa between 0.21-0.40, moderate when kappa between 0.41-0.60, good when kappa 0.61-0.80, and very good when kappa ≥0.80. Reported *P* values represent two-sided tests. Significance was defined as *P*<0.05.
